# Association Between Sleep Efficiency Variability and Cognition Among Older Adults: Cross-Sectional Accelerometer Study

**DOI:** 10.2196/54353

**Published:** 2024-04-04

**Authors:** Collin Sakal, Tingyou Li, Juan Li, Can Yang, Xinyue Li

**Affiliations:** 1 School of Data Science, City University of Hong Kong, Hong Kong, China (Hong Kong); 2 Center on Aging Psychology, Key Laboratory of Mental Health, Institute of Psychology, Chinese Academy of Sciences, Beijing, China; 3 Department of Mathematics, The Hong Kong University of Science and Technology, Hong Kong, China (Hong Kong)

**Keywords:** aging, cognition, accelerometer, sleep, sleep efficiency, geriatrics, gerontology, actigraphy, digital health, mhealth, cognitive impairments, mobile health, efficiency, variability, older adult, older adults, elder, elderly, older person, sleep quality, machine learning, quality of sleep, sleep

## Abstract

**Background:**

Sleep efficiency is often used as a measure of sleep quality. Getting sufficiently high-quality sleep has been associated with better cognitive function among older adults; however, the relationship between day-to-day sleep quality variability and cognition has not been well-established.

**Objective:**

We aimed to determine the relationship between day-to-day sleep efficiency variability and cognitive function among older adults, using accelerometer data and 3 cognitive tests.

**Methods:**

We included older adults aged >65 years with at least 5 days of accelerometer wear time from the National Health and Nutrition Examination Survey (NHANES) who completed the Digit Symbol Substitution Test (DSST), the Consortium to Establish a Registry for Alzheimer’s Disease Word-Learning subtest (CERAD-WL), and the Animal Fluency Test (AFT). Sleep efficiency was derived using a data-driven machine learning algorithm. We examined associations between sleep efficiency variability and scores on each cognitive test adjusted for age, sex, education, household income, marital status, depressive symptoms, diabetes, smoking habits, alcohol consumption, arthritis, heart disease, prior heart attack, prior stroke, activities of daily living, and instrumental activities of daily living. Associations between average sleep efficiency and each cognitive test score were further examined for comparison purposes.

**Results:**

A total of 1074 older adults from the NHANES were included in this study. Older adults with low average sleep efficiency exhibited higher levels of sleep efficiency variability (Pearson *r*=−0.63). After adjusting for confounding factors, greater average sleep efficiency was associated with higher scores on the DSST (per 10% increase, β=2.25, 95% CI 0.61 to 3.90) and AFT (per 10% increase, β=.91, 95% CI 0.27 to 1.56). Greater sleep efficiency variability was univariably associated with worse cognitive function based on the DSST (per 10% increase, β=−3.34, 95% CI −5.33 to −1.34), CERAD-WL (per 10% increase, β=−1.00, 95% CI −1.79 to −0.21), and AFT (per 10% increase, β=−1.02, 95% CI −1.68 to −0.36). In fully adjusted models, greater sleep efficiency variability remained associated with lower DSST (per 10% increase, β=−2.01, 95% CI −3.62 to −0.40) and AFT (per 10% increase, β=−.84, 95% CI −1.47 to −0.21) scores but not CERAD-WL (per 10% increase, β=−.65, 95% CI −1.39 to 0.08) scores.

**Conclusions:**

Targeting consistency in sleep quality may be useful for interventions seeking to preserve cognitive function among older adults.

## Introduction

Healthy sleep habits protect memory and cognitive function [1,2]. Sleep quality deteriorates with age, but older adults with cognitive impairments have worse sleep quality than their counterparts without impairments [3-5]. Lower sleep efficiency, a proxy for sleep quality, is associated with worse cognition among older adults [6]. The importance of getting sufficiently high-quality sleep to reduce individual risk of cognitive impairments has been reported [3,7]; however, the relationship between consistent sleep quality and cognition remains understudied. Because it is unreasonable to assume that older adults strictly adhere to a consistent sleep schedule on a nightly basis, the relationship between day-to-day sleep efficiency variability and cognition must be examined.

This cross-sectional accelerometer study aimed to quantify associations between sleep efficiency variability and performance on 3 cognitive tests assessing memory, categorical verbal fluency, and sustained attention while adjusting for demographic factors, chronic diseases, smoking habits, alcohol consumption, cardiovascular risk factors, depressive symptoms, and measures of activities of daily living (ADL) and instrumental activities of daily living (IADL). We additionally fit models using average sleep efficiency metrics to compare any observed relationships between sleep efficiency variability and cognition to those between average sleep efficiency and cognition.

## Methods

### Data Source and Study Design

Data from the US National Health and Nutrition Examination Survey (NHANES) 2011-2014 waves [8] were used, during which a subset of participants wore an ActiGraph GT3X+ device that objectively measured activity levels over 7 consecutive days immediately after all nonaccelerometer data were collected. Participants aged >60 years were also administered cognitive tests during the 2011-2014 waves. We excluded participants aged <65 years, without complete cognitive test data, or without at least 5 days of accelerometer wear time.

### Ethical Considerations

All NHANES participants provided informed consent, and ethics approval was granted by the National Center for Health Statistics Research Ethics Review Board (protocol #2011-17).

### Measuring Cognition

The NHANES 2011-2014 waves include 3 cognitive tests: the Digit Symbol Substitution Test (DSST), the Consortium to Establish a Registry for Alzheimer’s Disease Word-Learning subtest (CERAD-WL), and the Animal Fluency Test (AFT; Table 1) [9]. For the AFT, CERAD-WL, and DSST, higher scores correspond to better cognition.

**Table 1.** The 3 cognitive tests included in the 2011-2014 waves of the US National Health and Nutrition Examination Survey.

Cognitive test	Description
DSST^a^	Tests processing speed, sustained attention, and working memory. Scores range from 0 to 133 where 1 point is awarded for each symbol correctly written below its corresponding number based on a key mapping the symbols to the numbers.
CERAD-WL^b^	Measures immediate and delayed word recall. Three rounds of immediate recall and 1 round of delayed recall using lists of 10 unrelated words comprise the CERAD-WL. Scores on the CERAD-WL correspond to the number of correctly recalled words across all 3 rounds.
AFT^c^	Measures verbal categorical fluency and requires participants to name as many animals as possible in a 1-minute period.

a DSST: Digit Symbol Substitution Test.

b CERAD-WL: Consortium to Establish a Registry for Alzheimer’s Disease Word-Learning subtest.

c AFT: Animal Fluency Test.

### Deriving Sleep Metrics

Sleep efficiency—a proxy for sleep quality—is the ratio of time asleep to the total time between sleep onset and final sleep offset; possible values range 0-1 with higher values corresponding to better-quality sleep. Nightly sleep efficiency values were derived using an unsupervised hidden Markov model that identifies sleep-wake states using a data-driven machine learning approach [10]. The hidden Markov model algorithm has been validated against gold-standard polysomnography with 85.7% accuracy, 99.3% sensitivity, and better performance than commonly used supervised algorithms [10]. Sleep efficiency variability was defined as the SD of sleep efficiency across all nights of valid accelerometer data. For comparison purposes, we further derived each participant’s average sleep efficiency.

### Additional Covariates

To account for potential confounders, we gathered each participant’s age, sex, education, marital status, household income, smoking habits, current alcohol consumption, depressive symptoms, measures of functional independence, history of heart attack, history of stroke, and diagnoses of arthritis, heart disease, and diabetes. Depressive symptoms were quantified using Patient Health Questionnaire-9 scores [11]. A functional independence score was derived by summing responses to 20 ADL and IADL questions. Participants were categorized as current, former, or never smokers and drinkers. An explicit explanation of how each covariate was defined can be found in Multimedia Appendix 1. Participants with missing data were excluded to enable a complete-case analysis.

### Statistical Analysis

Participant characteristics were reported using the means and SDs for numeric variables and counts and percentages for categorical variables. We first examined the relationship between mean and day-to-day sleep efficiency variability using the Pearson *r* correlation coefficient and a scatterplot. Thereafter, using cutoffs from previous studies [12], we plotted the distribution of sleep efficiency variability stratified by normal versus low (≥0.85 vs <0.85) sleep efficiency.

We first examined univariable associations between sleep efficiency variability and DSST, CERAD-WL, and AFT scores. Demographic models were adjusted for age, sex, education, marital status, and household income. Finally, the full models in this study were further adjusted for depressive symptoms, ADL and IADL scores, smoking habits, alcohol consumption, diabetes, arthritis, heart disease, history of stroke, and history of heart attack. All univariable, demographic, and full models were refit using average sleep efficiency instead of day-to-day variability for comparison purposes. A sensitivity analysis was then conducted where we excluded extreme outliers (observations ≤1st or ≥99th quantile) for both average sleep efficiency and day-to-day variability. Models with both average and sleep efficiency variability were also examined (Multimedia Appendix 2).

## Results

### Descriptive Statistics

**Figure 1.**
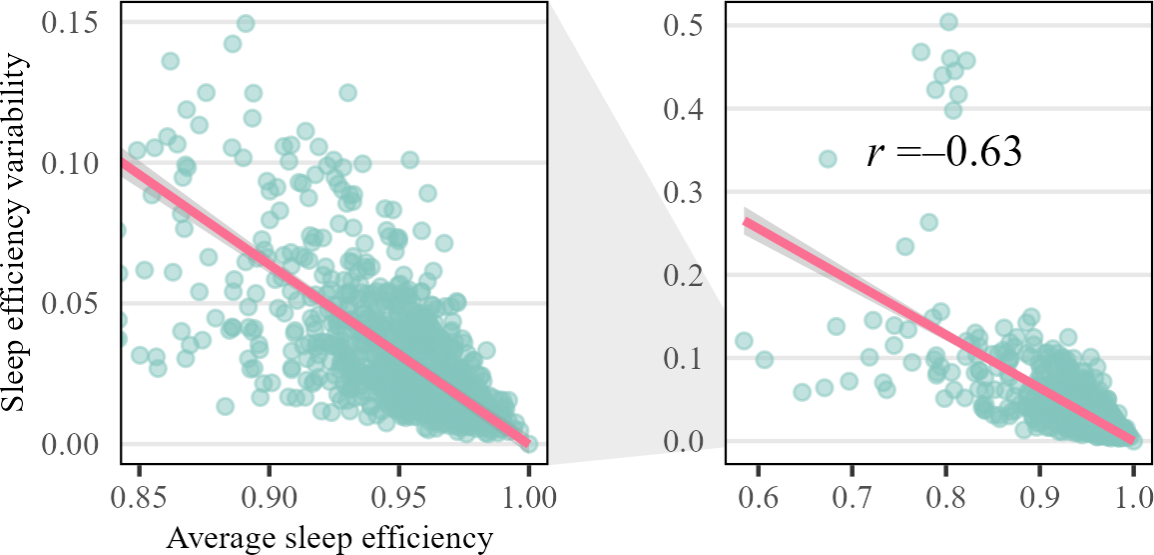
Scatterplot with a fitted regression line of average versus day-to-day variability for sleep efficiency.

**Figure 2.**
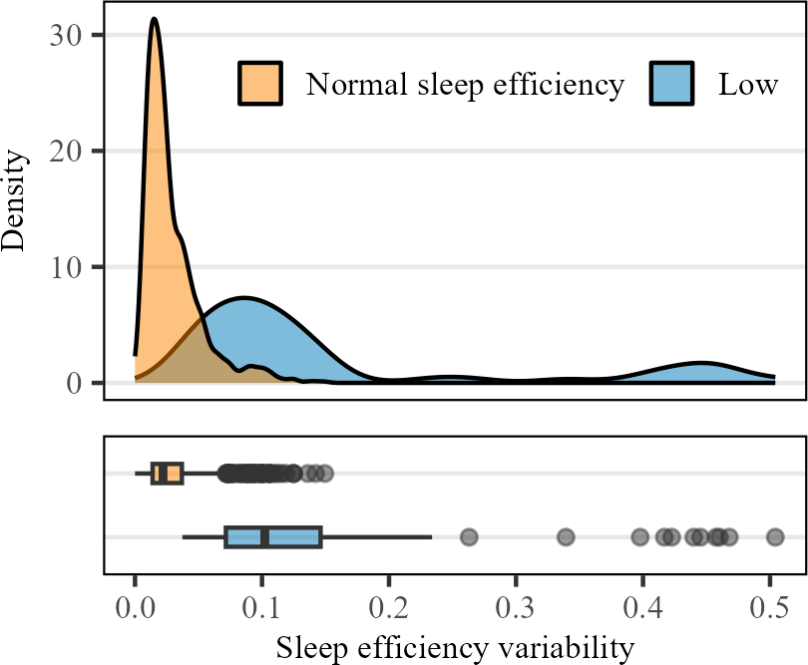
Distribution of day-to-day sleep efficiency variability stratified by average sleep efficiency.

In total, 1074 NHANES participants were included (mean age 72.3, SD 5.2 years; females: n=528, 49%), among whom 97.8% (n=1051) had ≥7 nights of sleep data (Table 2 and Table S1 and Figure S1 in Multimedia Appendix 1). The average sleep efficiency in the cohort was 0.94 (SD 0.05), while the average DSST, CERAD-WL, and AFT scores were 46.7 (SD 16.0), 25.0 (SD 6.29), and 16.8 (SD 5.25), respectively. The correlation between mean and day-to-day sleep efficiency variability was −0.63 (Figure 1). We found that older adults with low average sleep efficiency had higher levels of sleep efficiency variability than those with normal sleep efficiency levels (Figure 2).

**Table 2.** Demographic, sleep, and cognitive characteristics of older adults (N=1074) with valid accelerometer and cognitive test data who were part of the National Health and Nutrition Examination Survey.

Characteristic		Participants
Number of nights of sleep data, mean (SD)		7.8 (0.47)
Age (years), mean (SD)		72.3 (5.2)
**Sex, n (%)**		
	Male	546 (0.51)
	Female	528 (0.49)
**Education, n (%)**		
	Less than ninth grade	95 (0.09)
	Some high school	141 (0.13)
	High school graduate or GED^a^	245 (0.23)
	Some college or associate’s degree	307 (0.29)
	College graduate or above	286 (0.27)
**Marital status, n (%)**		
	Married	613 (0.57)
	Widowed	230 (0.21)
	Divorced	145 (0.14)
	Separated	19 (0.02)
	Never married	42 (0.04)
	Living with partner	25 (0.02)
Sleep efficiency variability, mean (SD)		0.04 (0.05)
Average sleep efficiency, mean (SD)		0.94 (0.05)
DSST^b^ score, mean (SD)		46.7 (16.0)
CERAD-WL^c^ score, mean (SD)		25.0 (6.29)
AFT^d^ score, mean (SD)		16.8 (5.25)

a GED: General Educational Development.

b DSST: Digit Symbol Substitution Test.

c CERAD-WL: Consortium to Establish a Registry for Alzheimer’s Disease Word-Learning subtest.

d AFT: Animal Fluency Test.

### Associations

In the univariable models, greater sleep efficiency variability was associated with lower scores on the DSST (per 10% increase, β=−3.34, 95% CI −5.33 to −1.34), CERAD-WL (per 10% increase, β=−1.00, 95% CI −1.79 to −0.21), and AFT (per 10% increase, β=−1.02, 95% CI −1.68 to −0.36). In the full models, greater sleep efficiency variability was associated with lower scores on the DSST (per 10% increase, β=−2.01, 95% CI −3.62 to −0.40) and AFT (per 10% increase, β=−.84, 95% CI −1.47 to −0.21) but not the CERAD-WL (per 10% increase, β=−.65, 95% CI −1.39 to 0.08; Tables 3-5). Conversely, greater average sleep efficiency was associated with higher scores on the DSST (per 10% increase, β=2.25, 95% CI 0.61-3.90) and AFT (per 10% increase, β=.91, 95% CI 0.27-1.56) but not the CERAD-WL (per 10% increase, β=.46, 95% CI −0.29 to 1.21) in the full models. In the sensitivity analysis, after excluding extreme averages and sleep efficiency variability outliers, all significant results observed in the full models remained significant (Multimedia Appendix 3).

**Table 3.** Associations between day-to-day variability and average sleep efficiency with Digit Symbol Substitution Test (DSST) scores.

Model covariates	Association with DSST scores	
	β (95% CI)^a^	*P* value
Sleep efficiency variability	−3.34 (−5.33 to −1.34)	.001
Mean sleep efficiency	4.28 (2.27 to 6.28)	<.001
Demographics + sleep efficiency variability	−2.04 (−3.69 to −0.39)	.02
Demographics + mean sleep efficiency	2.65 (0.97 to 4.32)	.002
Full model + sleep efficiency variability	−2.01 (−3.62 to −0.40)	.02
Full model + average sleep efficiency	2.25 (0.61 to 3.90)	.007

a Coefficients are reported per 10% increase.

**Table 4.** Associations of day-to-day variability and average sleep efficiency with Consortium to Establish a Registry for Alzheimer’s Disease Word-Learning subtest (CERAD-WL) scores.

Model covariates	Association with CERAD-WL scores	
	β (95% CI)^a^	*P* value
Sleep efficiency variability	−1.00 (−1.79 to –0.21)	.01
Mean sleep efficiency	0.85 (0.06 to 1.65)	.04
Demographics + sleep efficiency variability	−0.70 (−1.43 to 0.03)	.06
Demographics + mean sleep efficiency	0.52 (−0.23 to 1.26)	.18
Full model + sleep efficiency variability	−0.65 (−1.39 to 0.08)	.08
Full model + average sleep efficiency	0.46 (−0.29 to 1.21)	.23

a Coefficients are reported per 10% increase.

**Table 5.** Associations of day-to-day variability and average sleep efficiency with Animal Fluency Test (AFT) scores.

Model covariates	Association with AFT scores	
	β (95% CI)^a^	*P* value
Sleep efficiency variability	−1.02 (−1.68 to −0.36)	.002
Mean sleep efficiency	1.08 (0.42 to 1.74)	.001
Demographics + sleep efficiency variability	−0.85 (−1.48 to −0.22)	.009
Demographics + mean sleep efficiency	1.02 (0.38 to 1.66)	.002
Full model + sleep efficiency variability	−0.84 (−1.47 to −0.21)	.009
Full model + average sleep efficiency	0.91 (0.27 to 1.56)	.005

a Coefficients are reported per 10% increase.

## Discussion

### Principal Results and Comparisons With Prior Work

This study shows that older adults with higher sleep efficiency variability scored worse on the DSST and AFT after adjusting for demographic factors, chronic diseases, smoking habits, alcohol consumption, depressive symptoms, cardiovascular risk factors, and ADL and IADL scores. Effect sizes for average and sleep efficiency variability were similar in magnitude but in opposite directions, with greater variability being associated with lower DSST and AFT scores, while greater average sleep efficiency was associated with higher scores.

A previous accelerometer study found that greater sleep efficiency variability was associated with lower scores on serial subtraction tests and memory questionnaires [13]. However, the study was limited by a small sample (n<50) and did not consider relevant confounders such as chronic diseases, ADL and IADL, smoking habits, and alcohol consumption. Another study found that greater sleep efficiency variability was associated with a greater β-amyloid burden—a biomarker for Alzheimer disease—but was again limited by a small sample (n=52) [14]. Compared to existing studies, our work, using a larger cohort accounting for more confounders, provides evidence that greater sleep efficiency variability is associated with worse cognitive function among older adults. Furthermore, we found that effect sizes for sleep efficiency variability and average sleep efficiency were similar but in opposite directions, suggesting that getting sufficient and consistent high-quality sleep may be useful targets for interventions seeking to preserve cognitive function among older adults.

### Limitations

Given the cross-sectional nature of this study, we could not examine causal relationships. Bidirectional associations exist between certain forms of cognitive impairment and sleep disturbances [15], but they could not be examined herein. Future studies with longitudinal designs are therefore needed to further examine whether high sleep efficiency variability causally influences cognitive function or vice versa. Future studies may also benefit from collecting accelerometer data over longer durations, which more reliably measures sleep parameters [16], and from analyzing data across different countries to assess the generalizability of the findings presented herein. Lastly, polysomnography, the gold standard for measuring sleep parameters, was not used in this study. However, the use of wrist-worn accelerometers helped us assess sleep under natural living conditions, which intrusive polysomnography does not permit.

### Conclusions

Greater day-to-day sleep efficiency variability was associated with lower scores on 2 cognitive tests in this study. Our work may motivate future causal inference studies seeking to determine if consistency in sleep quality is a viable target for preserving cognitive function among older adults.

## Supplementary material

10.2196/54353 Multimedia Appendix 1 Participant flowchart, cohort chartacteristics, covariate definitions.

10.2196/54353 Multimedia Appendix 2 Additional models including both average and sleep efficiency variability

10.2196/54353 Multimedia Appendix 3 Sensitivity analysis.

## Abbreviations

ADL: activities of daily living
AFT: Animal Fluency Test
CERAD-WL: Consortium to Establish a Registry for Alzheimer’s Disease Word-Learning subtest
DSST: Digit Symbol Substitution Test
IADL: instrumental activities of daily living
NHANES: National Health and Nutrition Examination Survey
