# Supplementary material for: Association Between Sleep Efficiency Variability and Cognition Among Older Adults: Cross-Sectional Accelerometer Study
Source: JMIR Aging. 2024 Apr 4;7:e54353. doi: 10.2196/54353 (PMC11007383; doi:10.2196/54353)
Supplement: Multimedia Appendix 1 — Participant flowchart, cohort chartacteristics, covariate definitions. [file aging-v7-e54353-s001.docx]

Figure S1. Participant flowchart


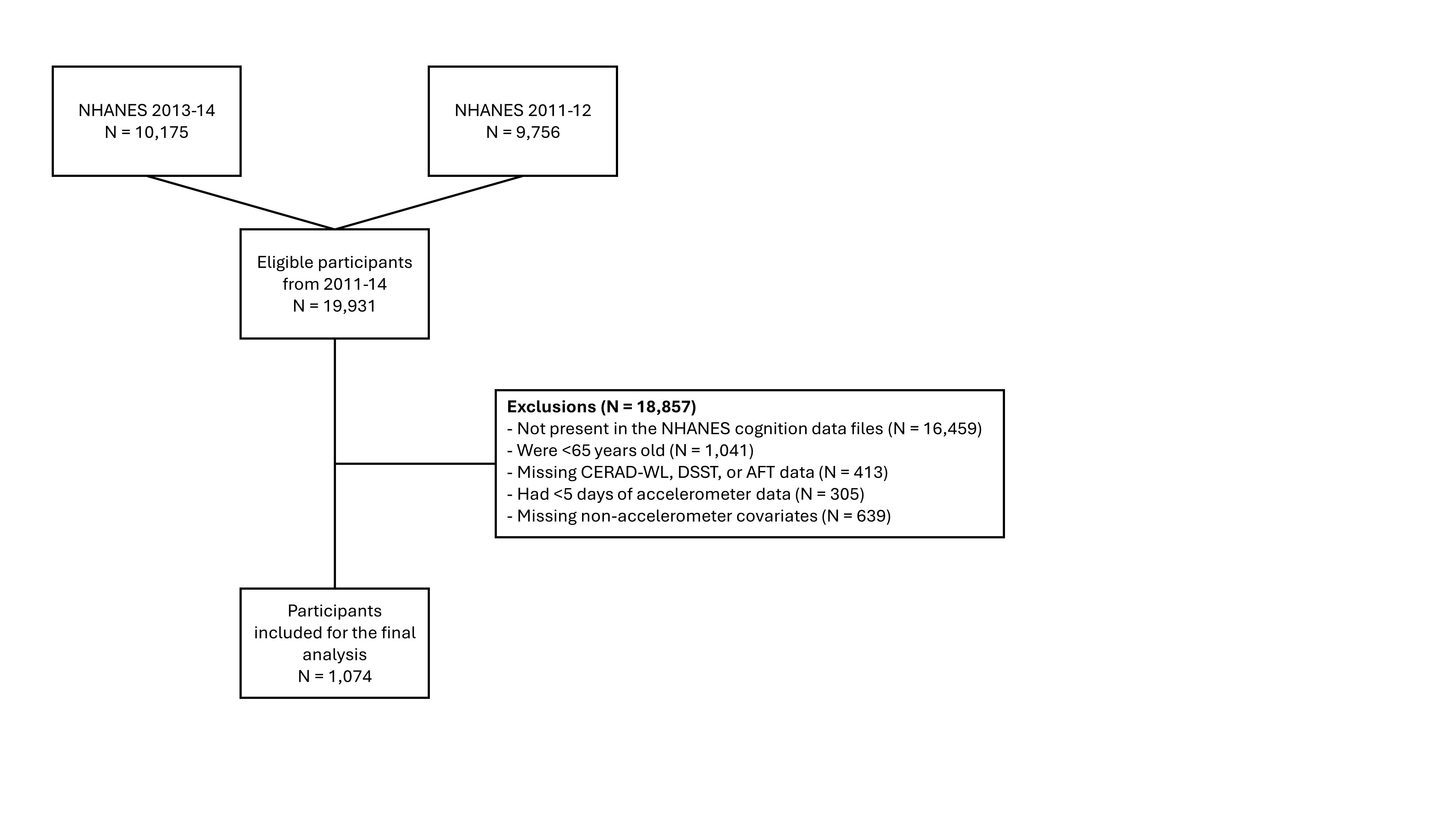


Table S1. Complete cohort characteristics

| Characteristic | | Participants (N = 1074) |
| --- | --- | --- |
| Age (years), mean (SD) | | 72.3 (5.2) |
| **Sex, n(%)** | |  |
|  | Male | 546 (0.51) |
|  | Female | 528 (0.49) |
| **Education, n(%)** | |  |
|  | Less than 9^th^ grade | 95 (0.09) |
|  | Some high school | 141 (0.13) |
|  | High school grad/GED | 245 (0.23) |
|  | Some college or associates degree | 307 (0.29) |
|  | College graduate or above | 286 (0.27) |
| **Marital status, n(%)** | |  |
|  | Married | 613 (0.57) |
|  | Widowed | 230 (0.21) |
|  | Divorced | 145 (0.14) |
|  | Separated | 19 (0.02) |
|  | Never married | 42 (0.04) |
|  | Living with partner | 25 (0.02) |
| **Household income (1000s of USD), n (%)** | |  |
|  | [0, 5) | 8 (0.01) |
|  | [5, 10) | 39 (0.04) |
|  | [10, 15) | 105 (0.10) |
|  | [15, 20) | 80 (0.07) |
|  | [20, 25) | 90 (0.08) |
|  | [25, 35) | 161 (0.15) |
|  | [35, 45) | 145 (0.14) |
|  | [45, 55) | 82 (0.08) |
|  | [55, 65) | 74 (0.07) |
|  | [65, 75) | 48 (0.04) |
|  | [75, 100) | 93 (0.09) |
|  | [100, Inf) | 149 (0.14) |
| **Diabetes, n (%)** | |  |
|  | Yes | 214 (0.20) |
|  | No | 860 (0.80) |
| **Arthritis, n (%)** | |  |
|  | Yes | 524 (0.49) |
|  | No | 550 (0.51) |
| **Heart Disease, n (%)** | |  |
|  | Yes | 112 (0.10) |
|  | No | 962 (0.90) |
| **Previous heart attack, n (%)** | |  |
|  | Yes | 90 (0.08) |
|  | No | 984 (0.92) |
| **Previous stroke, n (%)** | |  |
|  | Yes | 55 (0.05) |
|  | No | 0.95 (1019) |
| **Smoking status, n (%)** | |  |
|  | Current | 96 (0.09) |
|  | Former | 432 (0.40) |
|  | Never | 546 (0.51) |
| **Alcohol consumption status, n (%)** | |  |
|  | Current | 553 (0.51) |
|  | Former | 187 (0.17) |
|  | Never | 334 (0.31) |
| PHQ-9 score, mean (SD) | | 2.38 (3.36) |
| ADL/IADL score (functional independence), mean (SD) | | 22.7 (4.42) |
| DTDV^a^ sleep efficiency, mean (SD) | | 0.04 (0.05) |
| Average sleep efficiency, mean (SD) | | 0.94 (0.05) |
| DSST Score, mean (SD) | | 46.7 (16.0) |
| CERAD-WL score, mean (SD) | | 25.0 (6.29) |
| AFT score, mean (SD) | | 16.8 (5.25) |

Table S2. Definitions of derived covariates^a^

| **Variable** | **Derivation** |
| --- | --- |
| Household income | Was treated as an ordinal numeric variable in the models. Respondents who could only categorize their household income as above or below 20k USD/yr were treated as missing. |
| ADL/IADL score (functional independence score) | All “I don’t know” and “does not do this activity” were treated as missing in the ADL/IADL questions. NHANES variables used in the derivation were PFQ061A, PFQ061B, …., PFQ061T |
| Smoking status | Never – less than 100 cigarettes in their lifetime  Former – 100 cigarettes in their lifetime but does not smoke now  Current – smokes now |
| Alcohol status | Never – hasn’t had 12 drinks in any one-year period of their life  Former – has had 12 drinks in any one-year period but did not drink in the last 12 months  Current – has had 12 drinks in any one-year period in their life and has drank in the last year |

^a^This table includes information on variables that were encoded differently in this study than their default encoding in the NHANES data, or variables that were derived from multiple NHANES variables. For all variables in this study “I don’t know” and “Refused” responses were set to missing
