# Supplementary material for: Association Between Sleep Efficiency Variability and Cognition Among Older Adults: Cross-Sectional Accelerometer Study
Source: JMIR Aging. 2024 Apr 4;7:e54353. doi: 10.2196/54353 (PMC11007383; doi:10.2196/54353)
Supplement: Multimedia Appendix 2 — Additional models including both average and sleep efficiency variability [file aging-v7-e54353-s002.docx]

In the tables below we present models that include both average and day-to-day sleep efficiency variability. We encourage caution when interpreting the coefficients due to high correlation (Main Text Figure 1) between average sleep efficiency and day-to-day variability in sleep efficiency. Demographic models were adjusted for age, sex, education, marital status, and household income while full models were further adjusted for depressive symptoms, ADL/IADL scores, smoking habits, alcohol consumption, diabetes, arthritis, heart disease, history of stroke, and history of heart attack.

**Table 1.** Models with both average and day-to-day sleep efficiency variability for Digit Symbol Substitution Test (DSST) scores

|  | | Associations with DSST scores | | | |
| --- | --- | --- | --- | --- | --- |
|  |  | Average sleep efficiency | | Day-to-day sleep efficiency variability | |
| **Model Covariates** | | β (95% CI)^a^ | *P*-value | β (95% CI)^a^ | *P*-value |
|  | Unadjusted | 3.59 (0.99, 6.18) | 0.0068 | -1.08 (-3.65, 1.5) | 0.41 |
|  | Demographics | 2.21 (0.07, 4.36) | 0.043 | -0.68 (-2.79, 1.43) | 0.53 |
|  | Full model | 1.60 (-0.51, 3.70) | 0.14 | -1.03 (-3.1, 1.03) | 0.33 |

^a^Coefficients are reported per 10% increase

**Table 2.** Models with both average and day-to-day sleep efficiency variability for Consortium to Establish a Registry for Alzheimer’s Disease Word-Learning subtest (CERAD-WL) scores

|  | | Associations with CERAD-WL scores | | | |
| --- | --- | --- | --- | --- | --- |
|  |  | Average sleep efficiency | | Day-to-day sleep efficiency variability | |
| **Model Covariates** | | β (95% CI)^a^ | *P*-value | β (95% CI)^a^ | *P*-value |
|  | Unadjusted | 0.36 (-0.67, 1.38) | 0.50 | -0.77 (-1.79, 0.25) | 0.14 |
|  | Demographics | 0.12 (-0.84, 1.07) | 0.81 | -0.63 (-1.57, 0.31) | 0.19 |
|  | Full model | 0.07 (-0.89, 1.03) | 0.89 | -0.61 (-1.55, 0.33) | 0.20 |

^a^Coefficients are reported per 10% increase

**Table 3.** Models with both average and day-to-day sleep efficiency variability for Animal Fluency Test (AFT) scores

|  | | Associations with AFT scores | | | |
| --- | --- | --- | --- | --- | --- |
|  |  | Average sleep efficiency | | Day-to-day sleep efficiency variability | |
| **Model Covariates** | | β (95% CI)^a^ | *P*-value | β (95% CI)^a^ | *P*-value |
|  | Unadjusted | 0.71 (-0.14, 1.57) | 0.10 | -0.57 (-1.42, 0.28) | 0.19 |
|  | Demographics | 0.79 (-0.03, 1.61) | 0.058 | -0.36 (-1.17, 0.44) | 0.38 |
|  | Full model | 0.62 (-0.20, 1.44) | 0.14 | -0.46 (-1.27, 0.35) | 0.26 |

^a^Coefficients are reported per 10% increase
