# Supplementary material for: Association Between Sleep Efficiency Variability and Cognition Among Older Adults: Cross-Sectional Accelerometer Study
Source: JMIR Aging. 2024 Apr 4;7:e54353. doi: 10.2196/54353 (PMC11007383; doi:10.2196/54353)
Supplement: Multimedia Appendix 3 — Sensitivity analysis. [file aging-v7-e54353-s003.docx]

Table S1. Sensitivity analysis – associations between day-to-day variability and average sleep efficiency with Digit Symbol Substitution Test (DSST) scores (N = 1034)

| **Model Covariates** | **Association with DSST score** | |
| --- | --- | --- |
|  | β (95% CI)^a^ | *P*-value |
| Sleep efficiency variability | -10.0 (-13.7, -6.34) | <.001 |
| Mean sleep efficiency | 7.27 (4.61, 9.92) | <.001 |
| Demographics + sleep efficiency variability | -7.22 (-10.3, -4.17) | <.001 |
| Demographics + mean sleep efficiency | 4.96 (2.76, 7.16) | <.001 |
| Full model + sleep efficiency variability | -6.05 (-9.04, -3.05) | <.001 |
| Full model + average sleep efficiency | 4.20 (2.03, 6.37) | <.001 |

^a^Coefficients are reported per 10% increase

Table S2. Sensitivity analysis – associations between day-to-day variability and average sleep efficiency with Consortium to Establish a Registry for Alzheimer’s Disease Word-Learning subtest (CERAD-WL) scores (N = 1034)

| **Model Covariates** | **Association with CERAD-WL score** | |
| --- | --- | --- |
|  | β (95% CI)^a^ | *P*-value |
| Sleep efficiency variability | -1.61 (-3.07, -0.15) | .031 |
| Mean sleep efficiency | 1.19 (0.14, 2.24) | .027 |
| Demographics + sleep efficiency variability | -1.06 (-2.42, 0.30) | .128 |
| Demographics + mean sleep efficiency | 0.67 (-0.31, 1.65) | .180 |
| Full model + sleep efficiency variability | -0.87 (-2.24, 0.49) | .210 |
| Full model + average sleep efficiency | 0.61 (-0.37, 1.60) | .223 |

^a^Coefficients are reported per 10% increase

Table S3. Sensitivity analysis – associations between day-to-day variability and average sleep efficiency with Animal Fluency Test (AFT) scores (N = 1034)

| **Model Covariates** | **Association with AFT score** | |
| --- | --- | --- |
|  | β (95% CI)^a^ | *P*-value |
| Sleep efficiency variability | -2.20 (-3.42, -0.98) | <.001 |
| Mean sleep efficiency | 1.83 (0.96, 2.71) | <.001 |
| Demographics + sleep efficiency variability | -1.92 (-3.09, -0.75) | .001 |
| Demographics + mean sleep efficiency | 1.74 (0.89, 2.58) | <.001 |
| Full model + sleep efficiency variability | -1.67 (-2.85, -0.50) | .005 |
| Full model + average sleep efficiency | 1.54 (0.69, 2.38) | <.001 |

^a^Coefficients are reported per 10% increase
